# Are Bleeding Events Valid as a Surrogate Endpoint for Mortality in Cardiovascular Clinical Trials?∗

**DOI:** 10.1016/j.jacadv.2023.100335

**Published:** 2023-05-26

**Authors:** Seng Chan You

**Affiliations:** Department of Biomedical Systems Informatics, College of Medicine, Yonsei University, Seoul, Korea

**Keywords:** bleeding, patient-centered care, surrogate

**Figure:**
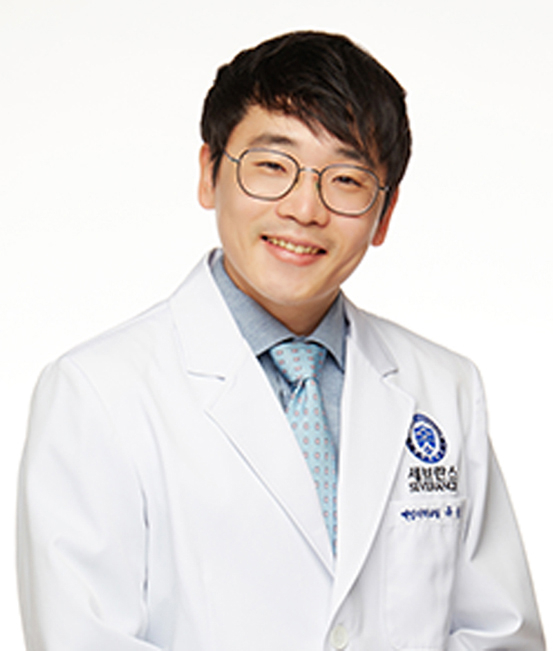

In contemporary medical practice, the ultimate objective is to provide patients with evidence-based medicine to achieve the best possible clinical outcomes. To determine which treatment strategy is most effective, randomized clinical trials are conducted.^1^ These trials are critical to ensuring that the treatments being delivered are safe, effective, and provide the desired outcome for patients. Traditionally, mortality has been the primary endpoint in cardiovascular trials.^2^, ^3^, ^4^, ^5^ However, as the field has evolved, composite endpoints have emerged as an effective means of evaluating treatment efficacy.^6^ Composite endpoints are a combination of death and several clinically relevant outcomes as surrogate endpoints, such as hospitalization or complications, which are assessed together to provide a comprehensive evaluation of the treatment strategy.^7^ The use of surrogate endpoints as primary composite endpoints in trials allows for the conduct of smaller trials that provide evidence for new treatments. While this approach has actively accelerated innovation in treatment strategy and helped to minimize futile megatrials for novel treatments,^8^ the concerns have also emerged.^9^

Recent cardiovascular clinical trials have tended to include major and/or minor bleeding events to the primary composite endpoints as net adverse clinical events,^10^^,^^11^ as bleeding during treatment with antithrombotic agents in patients with coronary artery disease (CAD) has been shown to be strongly associated with mortality.^12^ However, the application of net adverse clinical events as a measure raises concerns regarding its potential to produce null results due to the opposing directions of thrombotic and bleeding outcomes.^13^^,^^14^ Moreover, the validity of using these bleeding events as a surrogate endpoint for all-cause or cardiovascular death remains uncertain. In this issue of *JACC: Advances*, Kuno et al^15^ assessed the trial-level correlations between nonfatal bleeding events and mortality.

The study, which analyzed 48 randomized controlled trials with 181,951 participants, found that the trial-level R^2^ for major or minor bleeding were only 0.09 [95% CI: 0.00-0.26] and 0.09 [95% CI: 0.00-0.27] for all-cause or cardiovascular death, respectively. Even when confined to major bleeding, the R^2^ was only 0.03 (95% CI: 0.00-0.13) and 0.01 (95% CI: 0.00-0.05) for all-cause and cardiovascular death, respectively. Subgroup analyses by definitions of bleeding, study year, and follow-up duration did not show any significant correlations. These findings suggest that while bleeding events are important safety endpoints, they may not be a valid surrogate for mortality in clinical trials investigating antithrombotic agents for patients with CAD.

Of note, this study also discovered strong correlations between all-cause and cardiovascular mortality with Thrombolysis In Myocardial Infarction (TIMI) major or minor bleeding from 4 trials conducted in East Asia (R^2^ = 0.96 [95% CI: 0.81-1.00] and R^2^ = 0.98 [95% CI: 0.92-1.00]). This correlation may be related to the East Asian paradox, which refers the unique features of this ethnic group’s response to antiplatelet agents.^16^ Despite having a higher on-treatment platelet reactivity compared to the Western population, East Asians have a lower rate of ischemic events and a higher rate of bleeding events during antiplatelet therapy after percutaneous coronary intervention. Additionally, in study with follow-up period >12 months, R^2^ is 0.76 (95% CI: 0.61-1.00) and R^2^ is 0.89 (95% CI: 0.67-1.00) between all-cause mortality and TIMI major or minor bleeding in all patients as well as patients with acute coronary syndrome, while majority of included trials had shorter follow-up duration. This suggests that the possibility of a long-term correlation between bleeding and mortality cannot be excluded.

Historically, medicine and medical evidence has primarily revolved around the physician’s perspective.^17^ The ultimate objective of medical practice, which is to enhance clinical outcomes, may not necessarily equate to prolonging survival alone. It is noteworthy that a previous survey has shown disagreement between patients and trialists regarding the weights assigned to individual components of major cardiovascular events. Patients placed equal or even greater importance on reducing myocardial infarction or stroke compared to mortality, while trialists were much more concerned about avoiding death.^18^ Patients with CAD deserve to be treated as individuals, not just as medical cases. It is crucial to acknowledge that they have the right to enjoy their lives after receiving appropriate treatment. While mortality is a crucial outcome in clinical trials and clinical practice, it may not fully capture the impact of ischemic or bleeding events on individuals' daily lives. Nuisance bleeding, while not life threatening, can still significantly affect an individual's quality of life and adherence to treatment.^19^ Therefore, it is essential to consider bleeding risk and nuisance bleeding from a patient-centric or individual-centric perspective even though it is not directly related with mortality. This involves assessing the impact of bleeding events on individuals' daily activities, such as work, social life, and leisure activities. Patient-reported or individual-reported outcomes, such as bleeding diaries and quality-of-life questionnaires, can provide valuable information on the impact of bleeding events on individuals' lives.

In conclusion, this study provides important insights into the use of bleeding events as a surrogate endpoint for mortality in cardiovascular clinical trials. It highlights the need for careful consideration of primary endpoints in future trials, to ensure that they are relevant and meaningful for patients and that they accurately capture the desired treatment effect. Ultimately, to provide optimal care for patients with CAD, we need to take a holistic approach that considers both clinical outcomes and individuals' quality of life with tailored treatment plans.

## Funding support and author disclosures

Dr You is a chief technology officer of PHI Digital Healthcare.
